# Contribution of TAT System Translocated PhoX to *Campylobacter jejuni* Phosphate Metabolism and Resilience to Environmental Stresses

**DOI:** 10.1371/journal.pone.0026336

**Published:** 2011-10-20

**Authors:** Mary Drozd, Dharanesh Gangaiah, Zhe Liu, Gireesh Rajashekara

**Affiliations:** Food Animal Health Research Program, Department of Veterinary Preventive Medicine, Ohio Agricultural Research and Development Center, The Ohio State University, Wooster, Ohio, United States of America; Universität Münster, Germany

## Abstract

*Campylobacter jejuni* is a common gastrointestinal pathogen that colonizes food animals; it is transmitted via fecal contamination of food, and infections in immune-compromised people are more likely to result in serious long-term illness. Environmental phosphate is likely an important sensor of environmental fitness and the ability to obtain extracellular phosphate is central to the bacteria's core metabolic responses. PhoX is the sole alkaline phosphatase in *C. jejuni*, a substrate of the TAT transport system. Alkaline phosphatases mediate the hydrolytic removal of inorganic phosphate (Pi) from phospho-organic compounds and thereby contribute significantly to the polyphosphate kinase 1 (*ppk1*) mediated formation of poly P, a molecule that regulates bacterial response to stresses and virulence. Similarly, deletion of the *tatC* gene, a key component of the TAT system, results in diverse phenotypes in *C. jejuni* including reduced stress tolerance and *in vivo* colonization. Therefore, here we investigated the contribution of *phoX* in poly P synthesis and in TAT-system mediated responses. The *phoX* deletion mutant showed significant decrease (P<0.05) in poly P accumulation in stationary phase compared to the wild-type, suggesting that PhoX is a major contributor to the inorganic phosphate pool in the cell which is essential for poly P synthesis. The *phoX* deletion is sufficient for a nutrient stress defect similar to the defect previously described for the Δ*tatC* mutant. Additionally, the *phoX* deletion mutant has increased resistance to certain antimicrobials. The Δ*phoX* mutant was also moderately defective in invasion and intracellular survival within human intestinal epithelial cells as well as in chicken colonization. Further, the Δ*phoX* mutant produced increased biofilm that can be rescued with 1 mM inorganic phosphate. The qRT-PCR of the Δ*phoX* mutant revealed transcriptional changes that suggest potential mechanisms for the increased biofilm phenotype.

## Introduction

*Campylobacter jejuni* is the most common cause of campylobacteriosis, one of the most common gastrointestinal diseases in humans in the US [1]. It is a food-borne, Gram-negative, motile microaerobic bacterium that causes disease by invading the epithelial cells of the host's colon through subversion of microtubule structures in the epithelial cells [2], [3]. Damage to these cells disrupts the normal absorptive function of the colon and causes disease [3], [4]. Disease progression typically consists of abdominal pain, watery or bloody diarrhea, and fever which persists up to a week [4], [5]. Most cases of campylobacteriosis are self-limiting, but medical costs and losses of productivity total more than 2.4 billion a year [6]. Additionally, approximately 1/1000 clinical cases may result in long term neurological defects, including Guillain-Barre syndrome [7], [8]. This is particularly significant in immune-compromised populations, which comprise an estimated 3.6% of the total population in the US, who are more likely to become ill from *Campylobacter* and also more likely to experience long term sequelae [9], [10]. Further, the elderly population, which is likely to represent more than 20% of the US population in the next few decades, are also at increased risk for food-borne illnesses; including *Campylobacter* [11]–[13]. Therefore *Campylobacter* infections may become both more prevalent and severe as the population ages, making understanding *Campylobacter* cell physiology and controlling this pathogen urgently relevant.

Despite its success as an endemic gastrointestinal pathogen, the keys to *Campylobacter*'s virulence remain enigmatic. Unlike other enteric bacterial pathogens, *Campylobacter* does not have the advantages of a Type III secretion system, toxins, or other conventional pathogenicity factors [14]. *Campylobacter* is not known to proliferate outside of the host, but does adapt quickly to environmental stressors including osmotic pressure, nutrient starvation and antibiotic exposure [15]. One of the factors necessary for stress-survival and successful colonization is the Twin Arginine Translocation (TAT) system [16]. The TAT system is an inner membrane translocase that transports proteins folded in the cytoplasm across the inner membrane. Although the TAT system is ubiquitous among bacteria and archea, it does not have any animal homologues [17].

Using *in silico* screens, we have previously hypothesized that *Campylobacter* transports over a dozen proteins through TAT system [16]. Several proposed TAT substrates have been confirmed by laboratory methods; including HydB, MrfB, and Cj0415, MrfA, TorA, CueA, FdhA, the YedY homologue Cj0379c, and PhoX [18]–[20]. Some of these proteins are thought to have a role in cell physiology including stress survival, and we have previously found that the *C. jejuni* Δ*tatC* mutant is unable to establish long term colonization in chickens [16]. The mechanism of this survival defect, however, has not been well-studied.

One of the proteins that has been identified as a TAT substrate is PhoA*^(cj)^*, the only alkaline phosphatase in *Campylobacter* species [21]. The PhoA*^(cj)^* requires transport into the periplasm to become active and there it provides *Campylobacter* with inorganic phosphate (Pi) through the hydrolysis of phosphate groups from more complex organophosphate molecules. Also, the regulation of phosphate sensing and alkaline phosphatase activity in *Campylobacter* species is thought to have evolved separately from those in the more commonly studied *Escherichia coli* and *Bacillus subtilis*, and lacks some of the features common to these bacteria, including auto-regulation [20]. The *Campylobacter* alkaline phosphatase is more similar to those found in *Vibrio cholera*, *Pseudomonas*, as well as marine and soil bacteria [22], [23]. This family of alkaline phosphatases (PhoX) are typified by cytoplasmic folding, divergent sequences with little homology, and functional dependence of Ca^2+^ in place of Mg^2+^ or Zn^2+^ [23]. Since recent research has shown persuasively that *Campylobacter* alkaline phosphatase has evolutionary and functional similarities to the PhoX family of alkaline phosphatases, here we will refer to PhoA*^(cj)^* as PhoX [21]–[23].

Since Pi in the environment is typically low, alkaline phosphatases are necessary for nutrient and ATP homeostasis [24]. Pi is necessary for the PPK1 mediated formation of poly P, which has an important role in both basic metabolism and stress response [25]. Poly P has been shown to insulate cells in an alkali environment and be necessary for long term growth and survival in *Salmonella* [25], [26]. Additionally, cellular phosphate levels are often used by bacteria as an indicator of environmental resources; cellular responses to phosphate levels are closely connected to the control of starvation response as well as flagella growth, quorum sensing, and the production of virulence factors [24], [27], [28]. Many aspects of the *Campylobacter* phosphate response are mediated by the two component system *phosR/S*, a homologue to the *E. coli phoR/B* system [20]. In a microarray comparison of phosphate-starvation response, wild-type cells showed transcriptional modification of over 200 genes [29]. While some of these genes are likely affected by secondary responses, it is clear that the PhosR/S regulon is likely similarly important to cellular metabolism. Genetically similar *phoX* genes found in *V. cholera* have been shown to be involved in environmental survival mechanisms including control of biofilm formation, adjustment of cyclic-di-GMP levels, aerobic and heat-stress tolerance, stringent response as well as flagella function [24], [30]–[32]. Inorganic phosphate is known to also regulate additional genes including *spoT*, which subsequently affects the global regulator ppGpp [33]. Therefore, we hypothesize that some of the basic stress response defects seen in the *Campylobacter* TAT knockout mutant are caused by the inhibition of PhoX transport. We also hypothesize that these phenotypes may be linked with the subsequent interruption of poly P and poly P mediated stress responses. For both a way to understand the underlying physiology of an important pathogen as well as potential investment into understanding a mechanism that can be exploited for therapeutic use, we determined the role of *phoX* in *C. jejuni* pathophysiology.

## Materials and Methods

### Ethics statement

Animal experiment was conducted according to the guidelines of Association for Assessment and Accreditation of Laboratory Animal Care International (AAALAC). The animal studies are approved by The Institutional Animal Care and Use Committee (IACUC), The Ohio State University, under the protocol number 2010A00000155. As we study complex host pathogen interactions that are applicable to human health, the use of laboratory animals is unavoidable and justifiable. Chickens were housed at the Food Animal Health Research Program Animal Care Facility. The facility is fully accredited by AAALAC and animals were supervised by our senior veterinarian Dr. J. Hanson. Infectious agents were administered using manual restraint for less than one minute to minimize distress. Chickens were euthanized by carbon dioxide inhalation, which is rapid and painless. This method is consistent with the recommendations of the panel on euthanasia of the American Veterinary Medical Association and by The Ohio State University Institutional Laboratory Animal Care and Use Committee.

### Bacterial strains, plasmids, and culture conditions

Bacterial strains and plasmids used in this study are listed in Table S1. *C. jejuni* strain 81–176 (WT), a highly invasive strain originally isolated from an outbreak associated with raw milk [34], was used to generate the *phoX* deletion mutant. *C. jejuni* strains were routinely grown on Mueller-Hinton broth (MH; Oxoid) microaerobically [(85% N_2_ (v/v), 10% CO_2_ (v/v) and 5% O_2_ (v/v)] in a DG250 Microaerophilic Workstation (Microbiology International) at 42°C. MH agar plates were supplemented with *Campylobacter* selective supplement (SR117E, Oxoid) when isolating *C. jejuni* from chicken feces and organs. For growth curve and stress survival assays, *C. jejuni* was grown microaerobically in MH broth with appropriate antibiotics at 42°C with shaking at 200 rpm. *E. coli* DH5α was used for plasmid propagation and cloning purposes and was routinely cultured on Luria-Bertani (LB) medium at 37°C overnight. Growth media was supplemented with appropriate antibiotics, chloramphenicol (20 µg ml^−1^ for *E. coli*; 10 µg ml^−1^ for *Campylobacter*), kanamycin (30 µg ml^−1^) and zeocin (50 µg ml^−1^), where necessary.

### Construction of the *phoX* deletion mutant

Cloning and other molecular biology techniques were performed according to Sambrook & Russell [35]. Oligonucleotides were designed using Vector NTI® software (Invitrogen, Carlsbad, CA) and commercially synthesized by Integrated DNA Technologies (Skokie, IL). All the oligonucleotides used in the present study are listed in Table S2. Masterpure® DNA purification kit and Fast-Link DNA ligation kit were purchased from Epicentre (Madison, WI). Restriction enzymes were purchased from Promega. QIAquick® PCR purification kit and QIAprep® spin mini prep kit for plasmid isolation were purchased from Qiagen (Valencia, CA). Zero background cloning vector pZErO-1 and *E. coli* DH5α competent cells were purchased from Invitrogen.

Deletion of the *phoX* gene (CJJ_0181) was achieved by double crossover homologous recombination using a suicide vector containing approximately 1 kb of homologous sequences on either side of the *phoX* gene as described previously [16]. Briefly, the *phoX* along with 1 kb flanking region on either side of the target gene was amplified by PCR using PhoX-F and PhoX-R primers from *C. jejuni* 81–176 genomic DNA. The amplified PCR product was ligated into pZErO-1 to generate plasmid pZero1-*phoX*. Inverse PCR was performed on pZero1-*phoX* using PhoX INV-F and PhoX INV-R primers to delete majority of the *phoX* coding sequence. Kanamycin cassette from pUC4K was then cloned into inverse PCR product, the resulting suicide vector designated, pZero1-Δ*phoX*, was electroporated into *C. jejuni* 81–176 as described [16], [36]. Recombinants were selected on MH agar plates containing kanamycin, kanamycin resistant colonies were streak purified and one such mutant designated Δ*phoX* was used for further studies. The deletion of the *phoX* gene was confirmed by PCR.

### Complementation of the *ΔphoX* mutant

The complementation of *ΔphoX* mutant was accomplished by the insertion of a wild type *phoX* using pRRC integration vector [37]. The coding region of *phoX* along with its ribosome binding site was amplified by PCR using primers PhoX COMP-F and PhoX COMP-R. Restriction site (*Bam*HI) was included in each primer to facilitate cloning. Following digestion with *Bam*HI , product was ligated into the pRRC vector. The resulting construct was electroporated into *C. jejuni* Δ*phoX*::*kan*, and putative complemented clones were recovered on plates containing kanamycin and chloramphenicol. Insertion of the *phoX* gene in the rRNA spacer region was confirmed by PCR and further the constitutive expression of *phoX* was confirmed by phosphatase assay. One of the confirmed complemented clone designated *phoX^c^* was used for further analysis.

### Alkaline phosphatase assay

Alkaline phosphatase activity was determined as previously described [20]. Briefly, the Δ*phoX*, Δ*ppk1*, Δ*tatC*, *phoX* ^c^, and WT 81176 strains were grown overnight on MH plates with appropriate antibiotic selection. The cultures were gently scraped, washed in minimal essential medium (MEM), resuspended in MEM and incubated at 42°C microaerobically with shaking for 2 hours. Cultures were then centrifuged for 10 minutes at 7000 g and supernatant was removed. Cells were gently washed with 50 mM MOPS buffer (pH 7.4) (Sigma), OD_600_ was measured. The cells were pelleted, supernatant was removed, and cells were resuspended in PNPP buffer with 2 mM PNPP (Sigma) and incubated at 37°C. Absorption at 550 nm and 420 nm were taken using a spectrophotometer and the phosphatase activity was calculated as described previously [38]. The average and standard deviation of three replicate samples for each strain were calculated and the experiment was repeated three times.

### Isolation/Detection of poly P

Poly P was extracted using glassmilk and quantified using toluidine blue O as described earlier [39]. Poly P was quantified from mid-log, and stationary phase cultures by measuring the absorbance ratio at 530 to 630 nm spectrophotometrically using appropriate concentrations of phosphorous standard (Sigma Aldrich). The experiment was performed a total of three times under rich media (MH) and minimal media (MEM) conditions.

### Nutrient downshift assay

The role of *phoX* in *C. jejuni* survival under nutrient downshift was assessed using MEM with glutamine (Gibco 11095) or without glutamine (Sigma, M2279) and in the presence or absence of 1 mM Pi (Inorganic Ventures) as described previously [40]. In experiments where Pi was added to the media, the final concentration was 1 mM. This amount is similar to a previous study by Wosten et al [20] where concentrations above 0.4 mM show greater than 90% inhibition of alkaline phosphatase activity. However alkaline phosphate is induced in MEM which has low Pi concentration. Therefore, here we used MEM and 1 mM Pi. Additionally 1 mM Pi concentration had no effect on *C. jejuni* growth [20]. Briefly, mid-log-phase cultures of the WT, Δ*phoX*, and *phoX* ^c^, strains were pelleted, washed twice and resuspended in MEM with or without glutamine or Pi, and the OD_600_ was adjusted to 0.05. The suspensions were then incubated microaerobically at 42°C with shaking at 200 rpm. At different time points, 100 µl of cultures were serially diluted (10-fold) in respective MEM media and plated onto MH agar in triplicate. The plates were incubated microaerobically, and the number of CFU ml^−1^ was calculated. The experiment was performed three times.

### Osmotic stress response assay

Survival of C. *jejuni phoX* deletion mutant in the presence of osmotic stress was tested as described previously [39], [40]. To assess the osmotic stress survival in liquid culture, bacterial strains were grown to mid-log phase, adjusted to an OD_600_ of 0.05 in MH broth with and without 0.25 M NaCl and incubated microaerobically at 42°C for 48 hours with shaking at 200 rpm. A 100 µl of the culture at different time points was serially diluted (10-fold) and plated on MH agar plates. The plates were incubated microaerobically and CFU were determined. To assess the strains ability to tolerate osmotic stress on a solid medium, the WT, *ΔphoX*, and *phoX^c^* strains were grown to mid-log phase, serially diluted (10-fold), and 10 µl of diluted culture was spotted onto MH agar plates containing either 0.17 M NaCl or 0.17 M NaCl and 1 mM Pi. Plates were incubated microaerobically at 42°C for 2 days, the growth of *C. jejuni* was visualized and photographed. The experiment was repeated three times.

### Oxidative Stress response assay

To determine oxidative stress response, WT, *ΔphoX*, and *phoX^c^* strains were grown overnight on MH agar with the appropriate antibiotics at 42°C under microaerobic conditions. Cells were harvested the next day and 100 µl of bacterial culture containing 5×10^8^ CFU ml^−1^ (OD_600_ of 0.5) were spread on MH plates with appropriate antibiotics in the presence or absence of 1 mM Pi. A 5 mm well was cut into the middle of each plate and filled with 30 µl of 20 mM paraquat or 0.3% H_2_O_2_. Plates were incubated for 24 and 48 hours under microaerobic conditions and the zone of inhibition was measured and photographed. The experiment was performed a total of three times.

### Motility and biofilm assays

The *C. jejuni* wild type 81–176, *ΔphoX*, *phoX^c^*, *Δppk1*, and *Δppk1^c^* strains were tested for motility in semisolid MH medium plates containing 0.4% agar in the presence or absence of 1 mM Pi. Cultures were grown on MH agar plate under microaerobic conditions at 42°C for 48 hours. Culture densities were adjusted to OD_600_ of 0.05 and 2 µl of each culture was stabbed onto the surface of the motility plate. Plates were incubated at 42°C under microaerophilic conditions. Motility phenotypes were assessed after 24, and 48 hours, following inoculation. The motility assay was performed three times.

Static biofilm formation was assessed in borosilicate tubes as described previously [40], [41]. Briefly, overnight grown cultures of *C. jejuni* strains were diluted in MH broth with or without Pi to an OD_600_ of 0.05. Two milliliters of diluted culture was incubated at 42°C microaerobically for 3 days without shaking. Biofilms were visualized by staining with 250 µl of 1% (w/v) crystal violet for 15 min, rinsed 3 times with double distilled water. After third rinse, vials were photographed then quantified by measuring the absorbance at 570 nm after dissolving in 2 ml DMSO for 24 hours. The biofilm assay was performed three times.

### Antimicrobial susceptibility testing

Susceptibility to azithromycin, ciprofloxacin, erythromycin, tetracycline, florfenicol, nalidixic acid, telithromycin, clindamycin, and gentamicin was determined by using Sensititre® susceptibility plates for *Campylobacter* (TREK Diagnostic Systems, West Sussex, UK). Briefly, one hundred microliters of log-phase grown cultures adjusted to an OD_600_ of 0.05 in MH broth was added to each well in the Sensititre® susceptibility plate and the wells were covered using the perforated adhesive seal. The plates were incubated microaerobically at 42°C for 24 hours and the minimal inhibitory concentration (MIC) was recorded. Results were read following the manufacturer's instructions and interpreted according to MIC interpretive guidelines by Clinical Laboratory Standards Institute. In addition, the following antimicrobials were also tested individually as described previously [16]; polymixin B, cholic acid, taurocholic acid, deoxycholic acid, and an antimicrobial peptide of chicken origin, fowlicidin-1. One hundred microliters of the cultures grown above were added to serially diluted (2-fold) antimicrobials in a 96 well microtitre plate, mixed and the plates were incubated microaerobically at 42°C for 24 hours. The MIC was determined as the lowest concentration showing complete inhibition of visible growth. The susceptibility testing was repeated 3 times and the mean MIC (µg ml^−1^) was calculated.

### Quantitative RT-PCR

Quantitative RT-PCR (qRT-PCR) was performed, targeting a key gene involved in stringent response, *spoT*; post transcriptional global regulator, *csrA*; poly P associated enzymes, *ppk1* and *ppk2*; phosphate metabolism, *phosR*, *pstS*, *pstC*; oxidative stress, *cjj0379*, *cjj1374*, *ahpC*, *sodB*, and the multidrug resistance efflux pump gene, *cmeC* [20], [42], [43], [44]. Total RNA was extracted from log-phase grown bacterial cultures using RNeasy Mini Kit (Qiagen). The RNA concentration and purity was determined using NanoDrop ND-1000 spectrophotometer (Wilmington, DE). cDNA synthesis was carried out using SuperScript® III First-Strand Synthesis SuperMix (Invitrogen). Gene specific primers were designed to amplify the above mentioned genes along with *rpoA* or 16SrRNA (internal controls) using Beacon Designer 7.0 (Palo Alto, CA). Primers were obtained commercially from IDT-DNA and are described in Table S2. qPCR was performed using SensiMixPlus® SYBR RT-PCR Kit (Quantace, Norwood, MA) in a Realplex^2^ Mastercycler (Eppendorf, Westbury, NY). The relative levels of expression of genes were normalized with either *rpoA* or 16SrRNA amplified from the corresponding sample. The difference in expression of the genes was calculated using the comparative threshold cycle (CT) method to yield fold-difference in transcript levels [45]. The qRT-PCR was performed in duplicates and assay was performed a total of four times.

### INT407 invasion and intracellular survival assay

Invasion and intracellular survival assays were performed as described previously [39], [46]. Each well of a 24 well tissue culture plate was seeded with 1.4×10^5^ INT407 cells in MEM with 10% fetal bovine serum (FBS) and incubated for 18 hours at 37°C with 5% CO_2_. C. *jejuni* strains were grown to mid-log phase in MH broth microaerobically, the cells were pelleted at 5,000 g for 10 min, washed twice with MEM containing 1% FBS, resuspended in MEM to an OD_600_ of 0.02 (1.5×10^7^ cells) and used for infection. INT407 cells were infected with a multiplicity of infection 1∶100 for invasion and intracellular survival assays. For infection, 1 ml of bacterial cell suspension was pipetted on to INT407 cells, centrifuged at 1000 g for 3 min and incubated for 3 hours. For determining invasion, after 3 hours of incubation with bacteria, cells were treated with gentamicin (150 µg ml^−1^) and incubated for additional 2 h. After 2 hours of incubation, the infected cells were rinsed with MEM three times, lysed with 0.1% (v/v) Triton-X 100, serially diluted in MEM and plated on MH agar in duplicate to determine CFU. To assess intracellular survival, following 2 hours of gentamicin treatment the infected cells were washed with MEM three times and covered with MEM containing gentamicin (10 µg ml^−1^) and incubated for 24 hours. After 24 hours of incubation, the infected cells were washed with MEM, lysed with 0.1% (v/v) Triton-X 100, serially diluted in MEM and plated on MH agar in duplicate to determine CFU. The invasion and intracellular assays were performed three times.

### Chicken colonization study

Chicken colonization studies were performed as described previously [40]. Briefly, day-old broiler chicks (n = 6 for each group) from a local hatching facility (Food Animal Health Research Program, OARDC, Wooster, OH) were inoculated orally with 10^3^, and 10^5^ CFU of the C. *jejuni* WT and Δ*phoX* strains in 200 µl of PBS (pH 7.4). After 7 days post-inoculation, the chicks were euthanized. The ceca and feces were collected aseptically, weighed, homogenized, serially diluted in PBS (pH 7.4) and plated on MH agar containing *Campylobacter* selective supplement. Plates were incubated at 42°C microaerobically and CFU per gram of tissues were determined.

### Statistical analysis

Statistical significance of data generated in this study was determined using one-way analysis of variance (ANOVA) followed by Tukey's HSD (Honestly Significant Difference) test or Student's t-test (paired 2-tailed). *P*≤0.05 (α level) was considered statistically significant.

## Results

### The *ppk1* is not a primary effector of alkaline phosphatase activity

**Figure 1:**
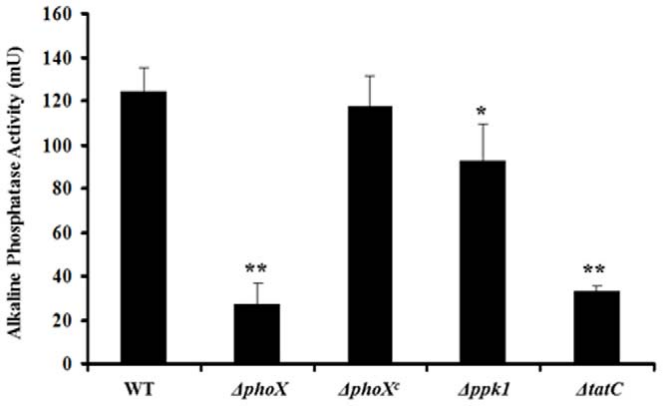
The *phoX* and *tatC* deletion mutants are defective in alkaline phosphatase activity. A PNPP based phosphatase assay was carried out in triplicate to determine alkaline phosphatase activity. The Δ*phoX* and the Δ*tatC* mutants show similar inhibition of alkaline phosphatase activity where as the *ppk1* mutant has alkaline phosphatase activity similar to wild type. Each data point is the mean ± standard deviation of 3 independent experiments. * *P*≤0.05. ** *P*≤0.01.

We found that Δ*phoX* mutant was significantly defective (P<0.01) in alkaline phosphatase activity (27.0 mU) compared to wild type (123.0 mU) (Fig. 1). Preliminary studies suggested that alkaline phosphatase activity could be measured with least background activity when cultures were incubated in MEM followed by washing with MOPS buffer before the assay (Fig. S1). Further, complementation of the Δ*phoX* mutant restored the alkaline phosphatase activity similar to wild-type levels (Fig. 1). Similarly, the Δ*tatC* mutant also showed similar alkaline phosphatase activity (33.5 mU) compared to the Δ*phoX* mutant (Fig. 1). This confirms previous results that PhoX is the only alkaline phosphatase in *C. jejuni* and it is solely transported through the TAT system [21]. The Δ*tatC* mutant, though not statistically significant, showed slightly increased alkaline phosphatase activity compared to the *phoX* deletion mutant.

It is well known that poly P levels are influenced by a wide range of stress responses, including low-nutrient stress [39]. Further, *spoT* deletion mutants have shown both diminished accumulation of poly P, decreased transcription of *ppk1* as well as increased transcription of *pstS* and *pstC*, which are regulated as part of the PhoR/S phosphate uptake regulon [39]. Therefore, we investigated whether there was a direct relationship between alkaline phosphate activity and poly P accumulation. The alkaline phosphatase level in the *ppk1* deletion mutant was 92.7 mU, slightly but significantly decreased compared to the wild-type (P<0.05) (Fig. 1). This suggests that *ppk1* expression does not severely affect alkaline phosphatase expression; they are potentially part of a separate, although intersecting function, of the phosphate utilization pathway.

### Poly P accumulation is reduced in both the *tatC* and *phoX* deletion mutants

**Figure 2:**
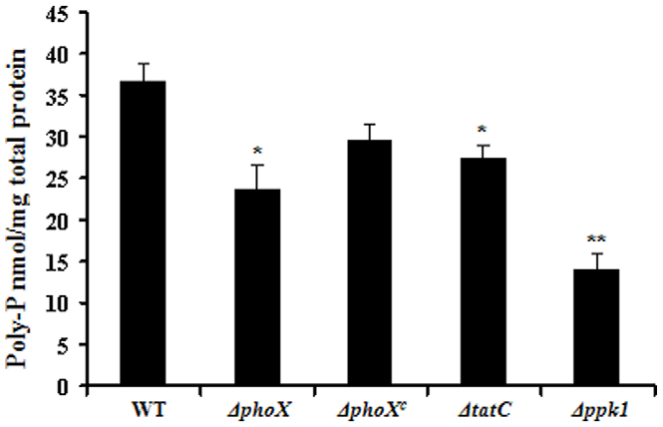
The *phoX* mutant is defective in poly P accumulation. Poly P was extracted from stationary phase grown wild type and *ΔphoX* mutant using glassmilk. The amount of poly P in the cell was determined by toluidine blue O method. Each data point is the mean ± standard error of 3 independent experiments. * *P*≤0.05. ** *P*≤0.01.

The *phoX* deletion mutant showed a statistically significant decrease (P<0.05) in poly P accumulation during stationary phase in the minimal media compared to the wild-type. The poly P levels of Δ*phoX* mutant were similar to the poly P levels of the *tatC* deletion mutant (Fig. 2). Both Δ*phoX* and Δ*tatC* had an average accumulated poly P level of approximately 21 nM poly P mg^−1^ of total protein and 25 nM poly P mg^−1^ of total protein respectively, compared to the wild-type cells (37 nM poly P mg^−1^ of total protein) (Fig. 2). However, *ppk1* deletion mutant showed a larger accumulation defect with only 14 nM poly P mg^−1^ total protein (P<0.01) (Fig. 2). Difference in poly P accumulation between *ppk1* and *phoX* mutants is likely due to ability of the *phoX* mutant to ameliorate the poly P defect with Pi obtained by other sources such as through phosphonate catabolism. *C. jejuni* has been shown to catabolize phosphonate [47], [48]. However, *ppk1* mutant can not synthesize poly P even in the presence of other Pi sources including phosphonates. This result suggests that interruption of *phoX* may be sufficient to cause the poly P defects seen in the *tatC* mutant as both Δ*tatC* and Δ*phoX* mutants showed similar poly P levels. Although, the Δ*tatC* mutant was defective in growth in rich media, the Δ*phoX* mutant grew similar to wild-type (data not shown, [21]). Similarly the poly P accumulation was significantly decreased in the *phoX* deletion mutant in the rich media during stationary phase (Fig. S2). There was no difference in the accumulation of poly P between mutant and the wild-type in the log phase (data not shown). Although alkaline phosphatase activity is necessary for *ppk1* mediated accumulation of poly P, the *ppk1* mutant had only a small defect in alkaline phosphatase activity. Alkaline phosphatase activity may be pleiotropically mediated by the function of *ppk1* or by other parts of the phosphate pathway under *ppk1* regulation.

### The *phoX* deletion results in sensitivity to nutrient but not osmotic and oxidative stress response

**Figure 3:**
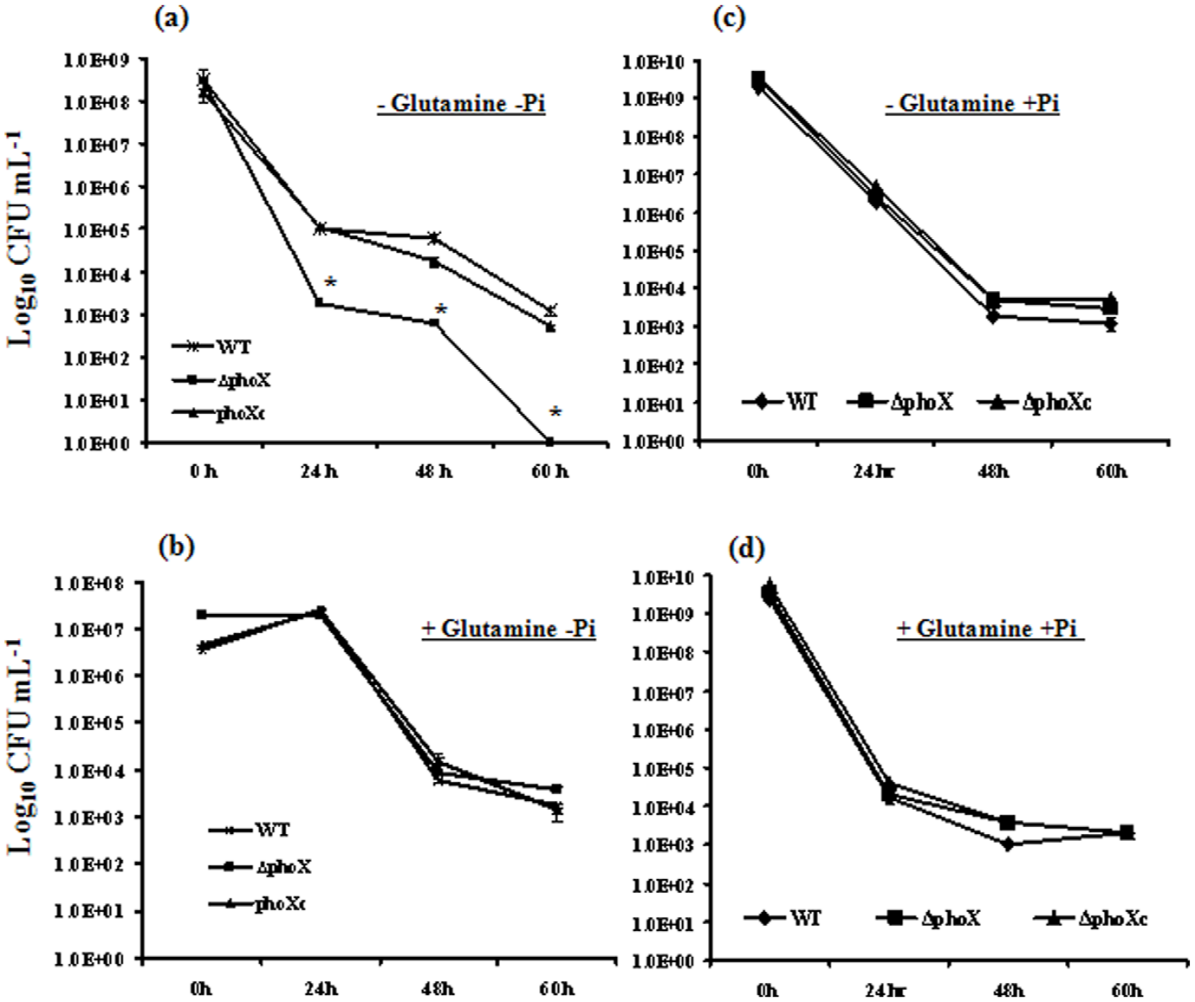
PhoX is necessary for survival under nutrient stress conditions. (**a**) Survival of *phoX* deletion, wild type, and the *phoX^c^* complemented strains under nutrient downshift was assessed by growing bacterial strains in minimal essential medium and determining the colony forming units at different time points. Addition of either 2 mM glutamine (**b**) or 1 mM Pi (**c**) or both (**d**) to nutrient downshift media improves *phoX* deletion mutant survival compared to wild type cells. Each data point represents the mean ± standard error of 3 independent experiments. * *P*≤0.05.

It is known that inorganic polyphosphate (poly P) is important to stress responses (nutrient, osmotic, osmotic and oxidative), and specifically the *C. jejuni ppk1* and *ppk2* mutants are sensitive to nutrient and osmotic stresses [25], [40], [46]. Since the Δ*phoX* mutant was defective in poly P accumulation; we investigated whether *phoX* deletion mutant is sensitive to various stresses. We found that the *phoX* deletion mutant had a significant defect (P<0.05) in nutrient stress survival after 24 hours of incubation in minimal media without glutamine (Fig. 3a). This survival defect was restored in the complemented strain (Fig. 3a). Similarly the *phoX* deletion mutant shows a statistically significant reduction in survival after 48 hours. The survival defect was even greater (approximately 3 logs), compared to wild type cells, at 60 hours (Fig. 3a).

Inorganic phosphate is required for deadenylation of glutamine synthetase, which is required for the synthesis of glutamine [49]. Since the *phoX* mutant is defective in Pi generation, we hypothesized that supplementation of glutamine might correct the nutrient downshift defect. The survival of the Δ*phoX* mutant was similar to WT in minimal media containing 2 mM glutamine even after 60 hours of incubation (Fig. 3b). Further, addition of 1 mM Pi to the media also corrected the survival defect of the Δ*phoX* mutant even the absence of glutamine (Fig. 3c). Since Pi is required for deadenylation of glutamine synthetase, which is required for synthesis of glutamine, the nutrient survival defect is likely due to insufficient Pi in the Δ*phoX* mutant.

The Δ*phoX* mutant had an osmotic stress and oxidative stress response similar to the wild-type strain both in the presence or absence of Pi (Fig. S3a–b). However, the qRT-PCR results indicated a downregulation of *katA* suggesting that mechanisms other than *katA* may play a role in oxidative stress response of *C. jejuni* [50]. Though the Δ*phoX* mutant showed consistently increased resistance to osmotic stress, it was not significant.

### The *phoX* deletion does not affect motility but has enhanced biofilm

**Figure 4:**
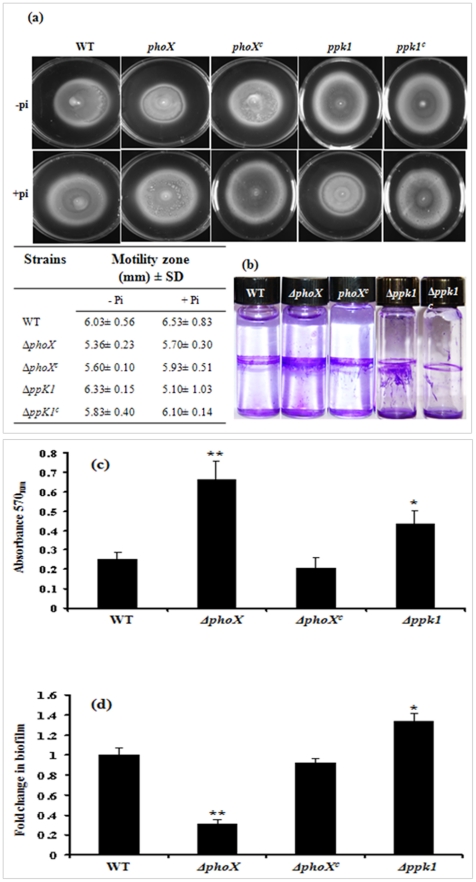
Motility and biofilm formation in the *ΔphoX* mutant. (**a**) Motility of the Δ*phoX* mutant is similar to the motility of the wild type strain and addition of Pi did not affect the motility. Values indicate average motility zone diameter ± standard error from 3 replicate experiments. Only representative images are shown. (**b**) The *ΔphoX* mutant shows enhanced biofilm formation. The biofilm was visualized by staining with 1% crystal violet for 15 min. (**c**) Quantification of biofilm using dimethy sulfoxide (DMS0). The amount of biofilm formed was dissolved in 2 ml DMSO for 24 hours and quantified by measuring absorbance at 570 nm. (**d**) Inorganic phosphate (1 mM) rescues the increased -biofilm phenotype in the Δ*phoX* mutants, but wild type biofilm is not changed by the addition of inorganic phosphate. The effect of 1 mM Pi on each strain was calculated as fold change in biofilm = (absorbance 570_nm_ with addition of 1 mM Pi)/(absorbance 570_nm_ without Pi added) Each bar represents the mean ± SD of 3 independent experiments. * *P*≤0.05. ** *P*≤0.01.

Poly P/inorganic phosphate stores have been linked to changes in both motility and biofilm [31], [40]. Additionally, regulation of Pi has been implicated in the ability of *V. cholera* to transit between marine and gastrointestinal lifestyles [30]. Therefore, we investigated whether *phoX* deletion would induce changes in motility and biofilm formation. The *phoX* deletion mutant did not show any motility defect on a semisolid agar. The motility of the *phoX* mutant was comparable to the wild type stain (Fig. 4a). Further, addition of Pi, though increased the motility, there was no significant difference between the WT and *ΔphoX* mutant. Similarly there was no significant difference in the motility of the *Δppk1* mutant compared to WT (Fig. 4a). On the other hand, the *phoX* deletion resulted in significantly enhanced biofilm formation (P<0.01) compared to the wild-type C. *jejuni* and the complementation of the *phoX* deletion restored biofilm to wild type levels (Fig. 4b–c). This result is surprising, since phosphate starvation in *V. cholera* and *Pseudomonas* is thought to reduce biofilm formation [30], [51].

Since *phoX* is closely associated with Pi levels, it is suggested that phosphate levels are an environmental indicator for biofilm regulation [30]. Therefore, we further tested whether biofilm formation was affected by Pi alone. We found that the *ΔphoX* mutant's increase of biofilm can be rescued with the addition of 1 mM Pi (P<0.01), strongly suggesting that the increase in biofilm is affected by the decreased availability of phosphate in the *phoX* deletion mutant (Fig. 4d). However, the wild-type strain did not show any changes in biofilm formation after the addition of 1 mM Pi (Fig. 4d), nor did the addition of 1 mM Pi affected the biofilm formation in the complemented strain (Fig. 4d). Consistent with our earlier finding, the Δ*ppk1* mutant also has an enhanced biofilm phenotype; however, addition of 1 mM Pi further increased the biofilm formation (P<0.05) in the *Δppk1* mutant (Fig. 4c–d) [40].

### The *phoX* deletion mutant has increased resistance to antimicrobials

Both the *ppk1* and *tatC* deletion mutants have been shown to have increased sensitivity to antibiotics [16], [40]. Since the *phoX* deletion mutant has a defect in poly P accumulation, we further investigated whether this would also result in susceptibility to antimicrobials. In contrast to *ppk1* and *tatC* deletion mutants, we found that the Δ*phoX* mutant has increased resistance to some common antimicrobials, including tetracycline, nalidixic acid and, to a lesser degree, to ciprofloxacin (Table 1). The *phoX* deletion mutant had 5, 4, 3-fold greater resistance to tetracycline, nalidixic acid, florfenicol and ciprofloxacin than wild-type (Table 1). The complementation with the wild type copy of the *phoX* restored susceptibility to wild-type levels (Table 1). Since biofilms have been implicated as a contributor to increased resistance to antibiotics, it is possible that the increased biofilm activity in the Δ*phoX* mutant is contributing to this phenotype. In previous studies, tetracycline, and nalidixic acid have been known to kill biofilm-forming cells less efficiently than non-biofilm forming cells [52]. This may suggest that the increased biofilm phenotype seen in *ΔphoX* may be a mechanism of innate increased antibiotic resistance.

**Table 1:** Antibiotic susceptibility of the Δ*phoX* mutant.

Antibiotic	Δ*phoX*: MIC ± SE (fold change)§	WT	*phoX* ^c^
zithromycin	0.05±0.02 (1.5)	0.03	0.03±0.02
Ciprofloxacin	0.19±0.08 (3.0)	0.06	0.06
Erythromycin	0.25±0.14 (1.4)	0.19±0.09	0.12±0.09
Gentamicin	1.50±0.58 (1.5)	1.0	1.0
Tetracycline	0.31±0.19 (5.2)	0.06	0.06±0.04
Florfenicol	1.50±0.58 (3.0)	0.50	1
Nalidixic Acid	16.0±9.2 (4.0)	4	4
Telithromycin	1.0±0.58 (2.0)	0.50	0.50
Clindamycin	0.38±0.19 (2.0)	0.19±0.09	0.19
Polymixin B	6.3 (0.5)	12.5	12.5
Cholic Acid	10,000 (2.0)	5,000	7,500±3.5
Taurocholic Acid	100 (1.2)	83.3±28.9	100
Deoxycholic Acid	25,000 (1.0)	25,000	25,000
Fowlicidin-1	16 (1.0)	16	16

§ fold change is the quotient of Δ*phoX*/wild type resistance for a given antibiotic.

All calculations are the average of three independent repetitions of the assay. Where standard error measurements are absent, measurement for all tests was same and standard error is zero. MIC; Minimal Inhibitory Concentration; SE, Standard Error.

### The *phoX* deletion results in transcriptional changes in key genes involved in phosphate uptake and stress responses

We used qRT-PCR to investigate how changes in alkaline phosphatase and inorganic phosphate starvation, caused by the *phoX* deletion, affected the transcription of genes that are commonly associated with environmental stress response or are believed to be part of the phosphate regulon. We found that *ppk1* was down regulated 2.1-fold in the *ΔphoX* mutant compared with a 7-fold down regulation of *ppk1* in the *tatC* mutant (Table 2). However, no changes in the transcription of *ppk2* were observed in the *phoX* deletion mutant although the *tatC* mutant showed 4-fold down-regulation (Table 2).

**Table 2:** qRT-PCR analysis of change in expression of selected genes in wild type, Δ*tatC*, and Δ*phoX* strains.

Gene/ORF	Fold change in Δ*phoX* compared to WT§	Fold change in Δ*tatC* compared to WT§
*ahpC (CJJ_0298)*	No change	−9.3
*ahpC (CJJ_0356)*	No change	−16.2
*CJJ_0379*	No change	−18.9
*ppk2*	No change	−4.4
*CJJ_1374*	−10.4	−15.7
*csrA*	2.6	No change
*katA*	−8.5	−19.2
*proP*	4.3	−27.5
*cmeC*	−5.8	No change
*pstC*	No change	Not tested
*pstS*	−2.4	Not tested
*ppk1*	−2.1	−7.1
*spoT*	−2.9	5.5
*sodB*	No change	−29.3

§ The difference in gene expression was determined by the threshold cycle(*CT*) method, and the assay was repeated three times with two replicates each time for each sample.

Data represent the mean relative fold change in expression.

WT, Wild Type; ORF, Open Reading Frame.

ProP has been known to confer osmotic protection [53]. The expression of *proP* was increased (4.3-fold) in the *phoX* deletion mutant, compared to a >25-fold down regulation in the *tatC* deletion mutant (Table 2). However, the *ΔphoX* mutant has a slightly increased, though not significant, resistance to osmotic stress (Fig. S3b–c) [16]. While *phoX* deletion increases *proP* expression, there may be other TAT-substrates which have additive and pleiotropic effects that result in the overall down-regulation of *proP* in the *tatC* deletion mutant. Also, we found that CJJ_1374, a VacJ homolog, known to be upregulated during oxidative stress, was down-regulated in both Δ*phoX* and Δ*tatC* mutants (Table 2) [43]. While CJJ_1374 was 1.5-fold more down-regulated in the *tatC* mutant than the *ΔphoX* mutant, the *tatC* mutant has an increased sensitivity to oxidative stress and the *ΔphoX* mutant does not (Table 2) [16]. Similarly, we saw an 8.5-fold down regulation of *katA* in the *ΔphoX* mutant, compared to a 19.2-fold down-regulation in the *tatC* mutant. Additionally, *cmeC* was down-regulated (5.8-fold) in the *ΔphoX* mutant. However, the *phoX* mutant was resistant to certain antimicrobials. It is possible that the antimicrobial resistance is mediated by changes other than CmeABC efflux pump, such as increased biofilm formation (Fig. 4; [54]).

We measured whether *pstS* and *pstC*, which are concurrently regulated by the PhosR/S regulon, would be transcriptionally affected by *phoX* deletion. We found a 2.4-fold down regulation of *pstS* and no change in the *pstC* gene (Table 2). This result was unexpected; we predicted that *pstS* would be upregulated during the putative phosphate depletion resulting from the deletion of *phoX*. This result may indicate that an indirect effect of *phoX* deletion, such as nutrient stress contributing to *pstS* regulation [55], [56]. We also observed a 2.9-fold down-regulation of *spoT*, a primary effecter of ppGpp degradation. In addition, a post transcriptional global regulator, *csrA* [24] was upregulated by 2.6-fold.

### Survival of the Δ*phoX* mutant is slightly diminished in INT407 cells

**Figure 5:**
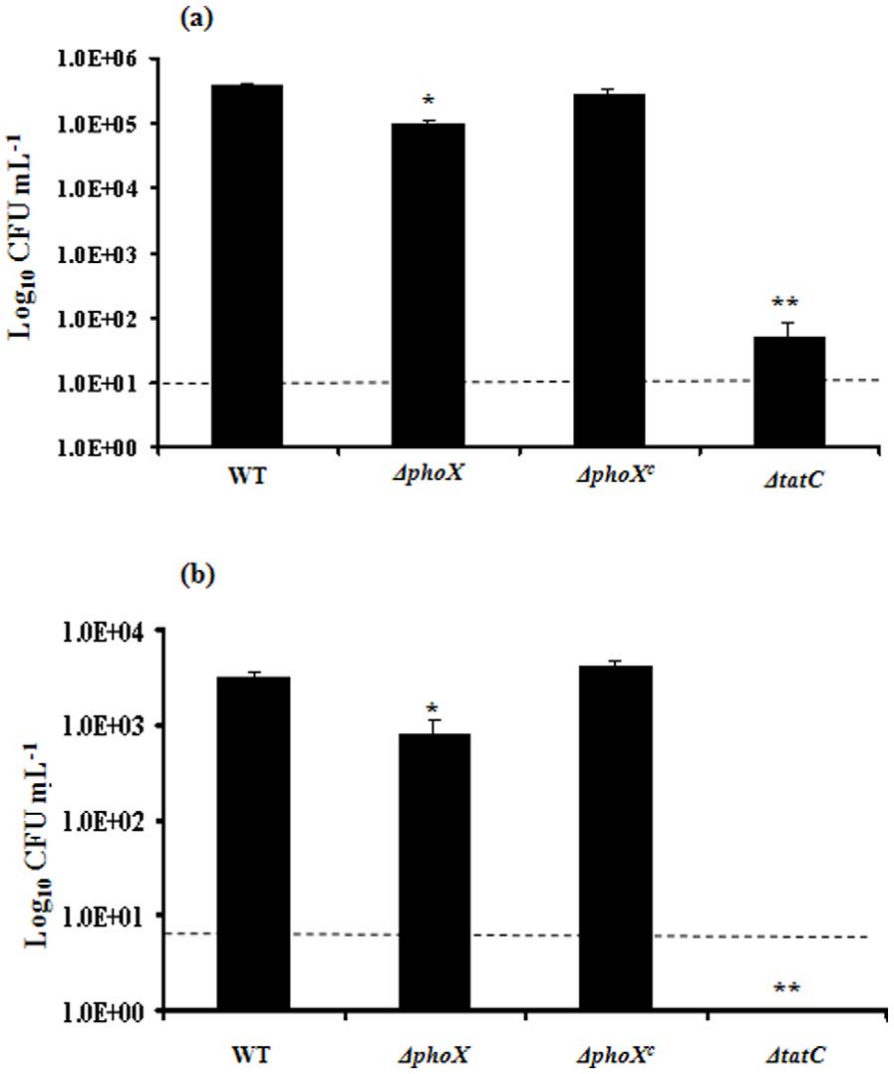
Invasion and intracellular survival of the *ΔphoX* mutant in INT407 cells. The *ΔphoX* mutant displays decrease in invasion (**a**) and intracellular survival (**b**) in INT407 human intestinal epithelial cells. Similarly, the Δ*tatC* mutant is also significantly deficient in invasion as well as intracellular survival in the INT 407 cells. The data represents the average of 3 experiments with 2 replicates in each experiment. Detection limit is represented by a dotted line. * *P*≤0.05.

To investigate if *phoX* is involved in virulence-associated phenotypes, we examined whether the *ΔphoX* mutant could invade and survive within INT407 human intestinal epithelial cells. The *phoX* deletion mutant was defective in invasion compared to wild-type cells by 4.4 fold (Fig. 5a). The *ΔphoX* mutant's intracellular survival was also similarly reduced by 4.0 fold compared to wild-type cells in INT407 cells while the complementation restored the defect to WT levels (Fig. 5a–b). However, the Δ*tatC* mutant had more than 7000-fold invasion defect in INT407 cells and no intracellular bacteria were recovered 24 hours post infection suggesting a severe intracellular survival defect (Fig. 5a–b). This suggests that *phoX* activity is not essential for successful invasion and intracellular survival; however, it should be noted that invasion and intracellular assays were performed using rich media (MEM supplemented with fetal bovine serum) where Pi was abundant and readily available even to the *ΔphoX* mutant. Since *ΔphoX* mutant, though defective in generation of Pi from complex organophosphate ester, is not defective in uptake of free Pi because it still carries intact Pi uptake *pst* genes [20], [24].

### The Δ*phoX* mutant shows diminished ability to colonize day-old chicks

**Figure 6:**
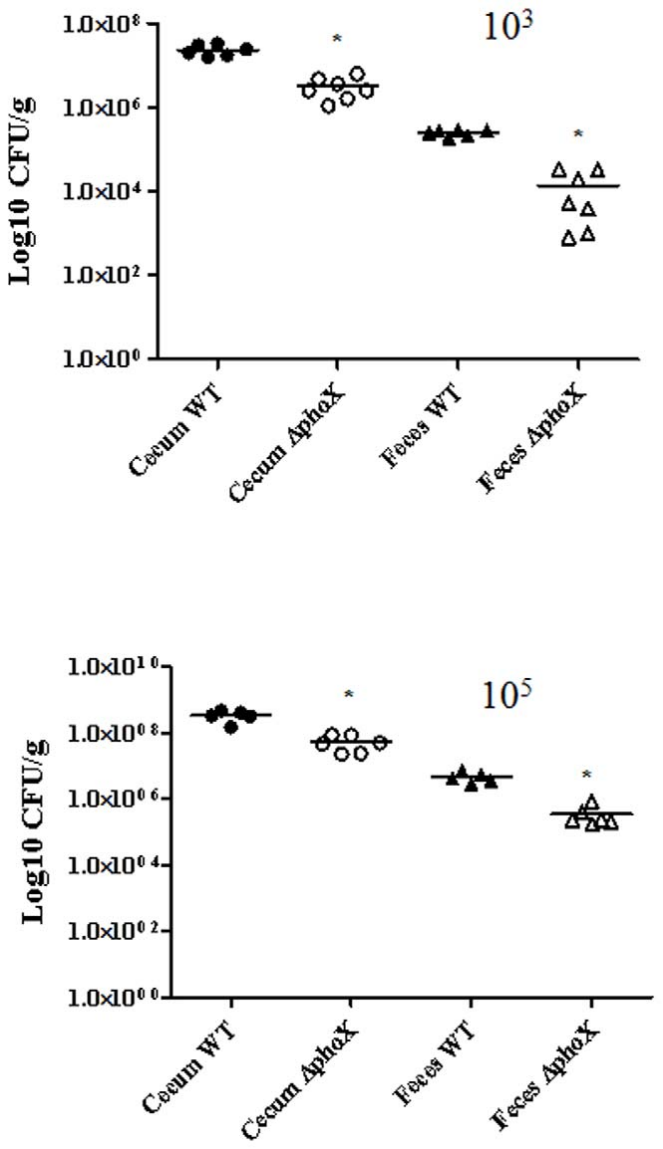
Colonization of the *ΔphoX* mutant in chickens. The *ΔphoX* mutant exhibits a dose-dependent colonization defect in day-old chicks. Eight days after inoculation, the chicks were sacrificed; cecum and feces were harvested and colonization level was assessed by determining the colony forming units/g of tissue. Each data point represents log_10_ colony forming units/g of tissue. * *P*≤0.05.

To assess the contribution of *phoX* to *C. jejuni* host colonization, we tested the ability of the *ΔphoX* mutant to colonize day-old chicks. We found that there was 0.8 log difference (P<0.05) in the number of wild-type (average CFU 2.34×10^7^ g^−1^) and *phoX* deletion mutant (average CFU 3.17×10^6^ g^−1^) bacteria recovered from the ceca 7 days after chicks were orally infected with 10^3^ CFU of each strain (Fig. 6). Similarly, there was approximately 1 log difference (P<0.05) in the number of C. *jejuni* wild-type (average CFU 3.41×10^8^ g^−1^) and *phoX* deletion mutant (average CFU 5.29×10^7^ g^−1^) recovered from chick ceca when the inoculation dose was 10^5^ CFU/chick (Fig. 6). Chicks inoculated with the *phoX* deletion mutant also had fewer bacteria in the feces compared to wild type. On an average there were approximately 1.1 and 1.2 log fewer bacteria in feces (P<0.05) in chicks inoculated with 10^3^ and 10^5^ CFU of the mutant (average CFU 1.38×10^4^; 3.53×10^5^ g^−1^), respectively, compared to chicks inoculated with similar amounts of the wild-type strain (average CFU 2.50×10^5^; 4.71×10^6^ g^−1^) (Fig. 6). These results suggest that there is a small but consistent defect in colonization caused by *phoX* deletion. It is possible that the effects of *phoX* deletion are ameliorated by using inorganic phosphate produced by alkaline phosphatases native to the chicks' gastrointestinal tract [52].

## Discussion

**Figure 7:**
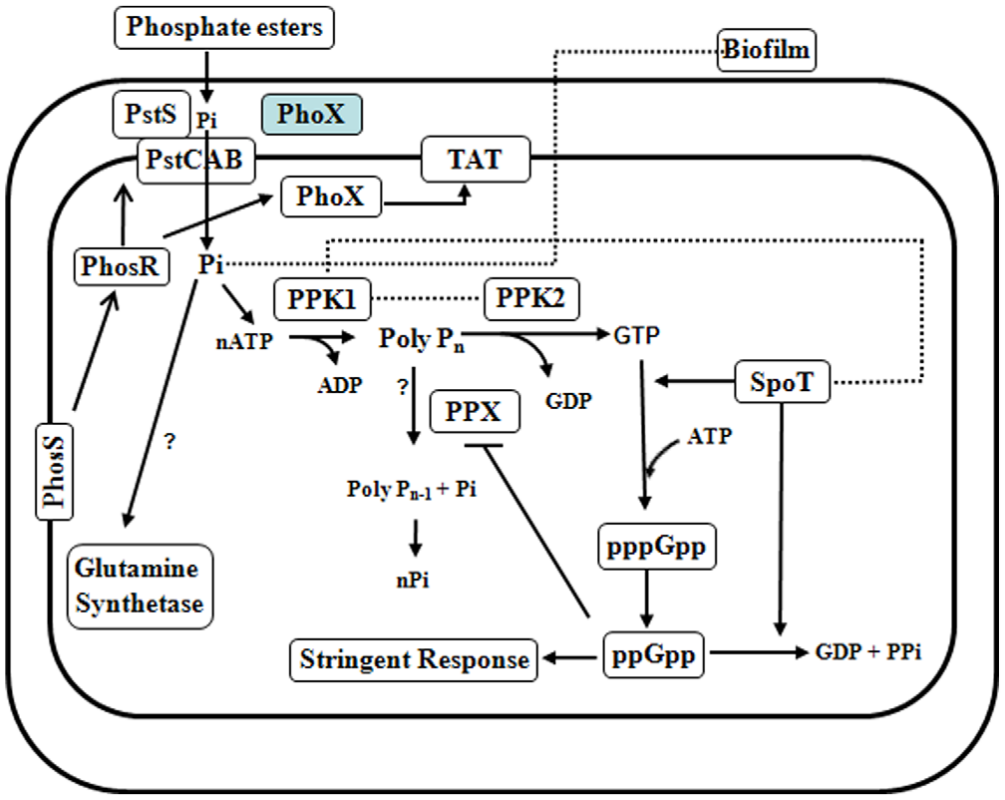
Hypothetical model illustrating interaction of different poly P-associated proteins in *C. jejuni*. Low phosphate conditions activate PhosS phosporylation of PhosR. Aklaline phosphatase (PhoX) is transcriptionally regulated by two component system PhosRS and is translocated by the TAT system to the periplasm where it is active. PhoX hydrolyzes organophosphate esters to release inorganic phosphate (Pi), which is a preferred source of phosphate for bacteria. Pi uptake into the cytoplasm is mediated by phosphate uptake proteins (PstSCAB) which are also directly regulated by PhosR. Pi is used directly by glutamine synthetase for glutamine metabolism. Also Pi appears to influence biofilm formation. ATP generated from Pi will be utilized for poly P synthesis by PPK1. PPK2 utilizes poly P to generate GTP, while PPX hydrolyzes poly P back to Pi. SpoT synthesizes pppGpp using GTP and ATP. pppGpp will be converted to ppGpp, a molecule that mediates stringent response in *C. jejuni* and other bacteria. SpoT also mediates hydrolysis of ppGpp to GDP and inorganic phosphate. PPK-1 may interact with SpoT to regulate its functions. Dashed lines and arrows indicate possible direct or indirect interaction of proteins. Question mark indicates potential pathways predicted based on the experimental evidence from other bacteria.

In this study we explored how one of the substrates of the TAT system, alkaline phosphatase, contributes to *C. jejuni* patho-physiology. Also we have expanded a model for poly P mediated responses in *C. jejuni* (Fig. 7) [39], [40], [46]. From our results, we can conclude the following about the phosphate metabolism of *C. jejuni*; alkaline phosphatase (*phoX*) is necessary for extracellular Pi acquisition and the effects of low inorganic phosphate are a significant part of stress metabolism through poly P and biofilm formation. In particular, *phoX* is important for survival in stationary phase in nutritionally limited conditions where it affects poly P metabolism. Further, without the ability to synthesize inorganic phosphate from extracellular sources *via* alkaline phosphatase, poly P accumulation is reduced. This suggests that extracellular sources of Pi are necessary for efficient poly P synthesis; *C. jejuni* is partially reliant on the environment to accumulate this important stress response and metabolic mediator. Since little is known about poly P regulation, understanding the origin of the inorganic phosphate that make up the poly P macro-molecule is novel.

Both the deletion of *tatC* and *ppk1* have been shown to have pleiotropic effects on bacteria including *C. jejuni* [16], [39], [40], [57]. Deletion of *tatC* can result in diverse phenotypes which can be a direct effect due to defect in translocation of necessary proteins or can be an indirect effect for which the mechanisms are not yet understood. Similarly, PPK1 has been linked with regulation of several genes in *P. aeruginosa*, *P. gingivalis* and *E. coli* [25], [58]. Specifically, microarray analysis of *ppk1* mutants in *P. aeruginosa* and *E. coli* revealed up-regulation of over 250 genes and down-regulation of more than 450 genes. Thus, poly P and its associated enzymes may be identified as “global regulators”. Our finding show that how the deletion of a single, specific variable (*phoX*) contributes to *C. jejuni* resilience to environmental stresses and provides an insight into understanding the complex mechanisms behind poly P and TAT system mediated stress responses.

The Δ*phoX* mutant has a 3 log nutrient stress defect that is rescued by the addition of 2 mM glutamine (Fig. 3), suggesting that the Δ*phoX* nutrient stress phenotype is caused by a defect in glutamine metabolism. Interestingly the nutrient downshift defect was also rescued by the addition of 1 mM Pi even in the absence of glutamine. Since it is known that inorganic phosphate is crucial for the deadenylation of glutamine synthetase [49], it is likely that the nutrient stress defect is associated with defective inorganic phosphate in the *ΔphoX* mutant. Further, it is reasonable to assume that defective translocation of alkaline phosphatase may be responsible for the nutrient stress phenotype seen in the Δ*tatC* mutant since both Δ*phoX* and Δ*tatC* mutants appear to have similar nutrient downshift defects (Fig. 3) [16].

The Δ*phoX* has an increased biofilm phenotype that can be rescued with the addition of 1 mM Pi (Fig. 4d). This suggests that reduced Pi concentration, and consequently reduced poly P, may be a factor that allows C. *jejuni* to sense an environment hostile to growth and initialize defensive measures. This could promote *C. jejuni* survival on surfaces—which are typically low phosphate environments—and improve the pathogen's ability to endure until it can reach a suitable host. The biofilm results may explain the *phoX* deletion mutant's decrease in sensitivity to tetracycline (Table 1), a drug that is known to have reduced penetration of biofilms [52].

The rescue of the biofilm phenotype by addition of 1 mM Pi (Fig. 4d) suggests that the biofilm phenotype is not a direct result of *phoX* deletion, but may be result of cellular response to the Pi depleted conditions that the *phoX* deletion mutant created. In contrast, in the Δ*ppk1* mutant biofilm formation was further increased with the addition of 1 mM Pi. Since, the *ppk1* mutant is defective in the synthesis of poly P, unlike the *phoX* mutant, the different outcome in the *ppk1* mutant in response to Pi is likely due to pleiotropic effects that the *ppk1* deletion has on the *C. jejuni* [39], [40]. This response suggests that although low levels of inorganic phosphate may be sufficient to increase biofilm, poly P and other factors modify this response. Since the Δ*ppk1* mutant showed increased biofilm in the presence of Pi, we hypothesize that there is a phosphate-related biofilm response parallel to the poly P mediated response and perhaps tied to other environmental stressors.

Our qRT-PCR data shows that a slight down-regulation of *ppk1* transcription in the Δ*phoX* mutant in the stationary phase. This result agrees with the poly P accumulation defect that we observed. Additionally, we saw that the Δ*tatC* mutant had a greater down-regulation of *ppk1* than Δ*phoX*, this may suggest that an additional TAT substrate contributes to poly P synthesis. Although we found that *phoX* deletion results in transcriptional down-regulation of oxidative stress genes (*katA*, *CJJ_0379*, *vacJ* homologue *CJJ_1374*, *ahpC*) these transcriptional changes did not result in reduced oxidative stress survival phenotype (Fig. S3). These genes are universally more down-regulated in the Δ*tatC* mutant where the mutant has a significantly increased sensitivity to oxidative stress [16]. However, when grown in minimal media, *phoX* deletion in general caused fewer transcriptional changes (data not shown); there was a 2.4-fold upregulation of *ppk1* and 3.5-fold decrease in *ppk2* transcription compared to wild-type. This may suggest that *ppk1* and *ppk2* regulation is pleiotropically affected by nutritional stressors.

We also conclude that *phoX* is unlikely to be more than a peripheral mechanism for the survival defect in INT407 cultured cells as well as *in vivo* chicken colonization. This agrees with previous research on *phoB* deletion mutant in chickens; the host's gastrointestinal surfaces have their own alkaline phosphatase enzymes, allowing the Δ*phoX* mutant to compensate for its alkaline phosphatase deficiency by uptake of host derived free phosphate [59], [60]. Less inorganic phosphate availability and other factors such as differences in temperature and oxygen levels in feces compared to ceca could explain an increased survival defect of Δ*phoX* mutant in feces compared to ceca.

In *C. jejuni* we found that deletion of *phoX* resulted in increased biofilm formation. However, in *V. cholera*, low intracellular phosphate results in decreased biofilm formation that is regulated by PhoB [59]. This is surprising because biofilm inhibition in *V. cholera* is thought to be part of a cellular transition to an aquatic lifestyle. Similar to *V. cholera*, *C. jejuni* contamination of water is a known source of infection [5], [59]. *C. jejuni*'s increase of biofilm in response to deletion of *phoX* and presumably diminished intracellular phosphate is more similar to plant and soil dwelling pathogens such as *A. tumefaciens* [61]; this may suggest that the phosphate stressor response that *C. jejuni* has evolved is more tuned towards survival on an exposed surface rather than a marine environment [62]. Although *V. cholera* and *Pseudomonas* have evolved similar *phoX* genes, there may be crucial phenotype differences that are likely unique to *C. jejuni* and have an impact on its cell physiology and survival [51], [59]. For instance, phosphate starvation in *Campylobacter* increases biofilm rather than decreasing it, and phosphate starvation did not increase oxidative stress sensitivity, or cause a change in motility [20], [63].

In summary, these finding reinforce an important, central theme to alkaline phosphatase and phosphate utilization in *C. jejuni*. It is nearly universal that the ability to use inorganic phosphate from extracellular environments is critical for bacterial physiology. The molecular response to this information however is highly dependent on the bacterial species and its environment. Therefore we hope that our study will advance understanding of the phosphate utilization in *Campylobacter* a little further and perhaps suggest additional possible combinations of cellular responses to those modeling other *phoX* containing bacteria.

## Supporting Information

Figure S1 ***C. jejuni*** **alkaline phosphatase activity in different culture media.** Background phosphatase activity in the *ΔphoX* mutant grown in different *Campylobacter* culture media. The background alkaline phosphatase activity in the *ΔphoX* mutant was least in minimal essential medium. Additional washing with MOPS buffer reduced variation and improved alkaline phosphatase activity in the wild type. Each data point is the mean ± standard deviation of 3 experiments. * *P*≤0.05.
(TIF)

Figure S2 **Poly P Accumulation in** ***ΔphoX*** **mutant grown in rich media.** The *phoX* mutant is defective in poly P accumulation. Using glassmilk, Poly P was extracted from stationary phase wild type, *ΔphoX, ΔphoX^c^*, *ΔtatC and Δppk1* strains grown in MH media. The amount of poly P in the cell was determined by toluidine blue O method. Each data point is the mean ± standard deviation of 3 independent experiments. * *P*≤0.05.
(TIF)

Figure S3 **Oxidative and osmotic stress tolerance of the** ***ΔphoX*** **mutant.** (**a**) The Δ*phoX* mutant has a similar zone of inhibition to the wild type strain when exposed to 20 mM paraquat or 0.3% H_2_O_2_ for 24 hours under microaerobic conditions. Addition of 1 mM Pi did not affect the oxidative stress response. Values indicate average zone of inhibition diameter ± standard deviation from three replicate experiments. Only representative images are show. (**b–c**) wild type, *ΔphoX*, and *phoX^c^* strains were grown to mid-log phase, osmotic stress tolerance was determined either on solid media (MH agar) containing 0.17 M NaCl (**b**) or in liquid media (MH broth) containing 0.25 M NaCl (**c**). These experiments were performed three times.
(TIF)

Table S1 **Bacterial strains and plasmids used in this study.**
(DOC)

Table S2 **Oligonucleotide primers used in this study.**
(DOCX)
