# Supplementary figures and images for: Contribution of TAT System Translocated PhoX to Campylobacter jejuni Phosphate Metabolism and Resilience to Environmental Stresses
Source: PLoS One. 2011 Oct 20;6(10):e26336. doi: 10.1371/journal.pone.0026336 (PMC3197622; doi:10.1371/journal.pone.0026336)

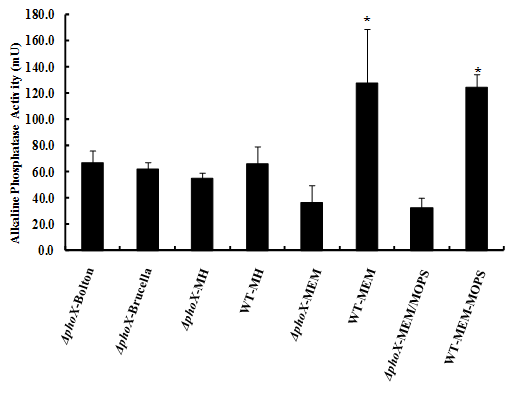

Supplement: Figure S1 — C. jejuni alkaline phosphatase activity in different culture media. Background phosphatase activity in the ΔphoX mutant grown in different Campylobacter culture media. The background alkaline phosphatase activity in the ΔphoX mutant was least in minimal essential medium. Additional washing with MOPS buffer reduced variation and improved alkaline phosphatase activity in the wild type. Each data point is the mean ± standard deviation of 3 experiments. * P≤0.05. (TIF) [file pone.0026336.s001.tif]

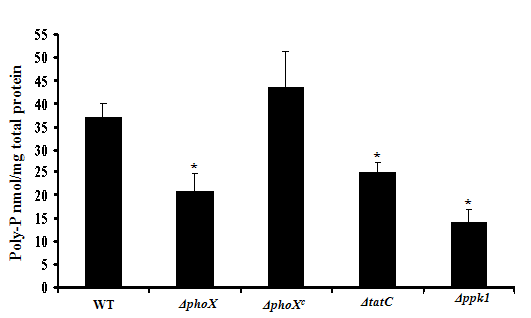

Supplement: Figure S2 — Poly P Accumulation in ΔphoX mutant grown in rich media. The phoX mutant is defective in poly P accumulation. Using glassmilk, Poly P was extracted from stationary phase wild type, ΔphoX, ΔphoXc, ΔtatC and Δppk1 strains grown in MH media. The amount of poly P in the cell was determined by toluidine blue O method. Each data point is the mean ± standard deviation of 3 independent experiments. * P≤0.05. (TIF) [file pone.0026336.s002.tif]

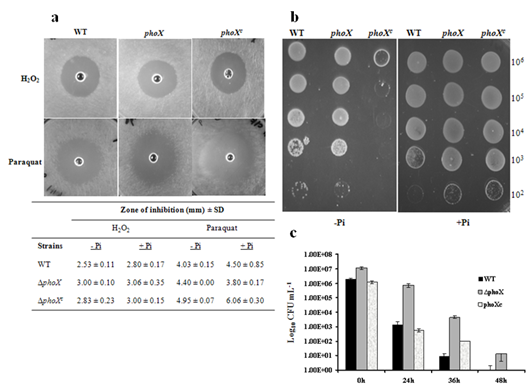

Supplement: Figure S3 — Oxidative and osmotic stress tolerance of the ΔphoX mutant. (a) The ΔphoX mutant has a similar zone of inhibition to the wild type strain when exposed to 20 mM paraquat or 0.3% H2O2 for 24 hours under microaerobic conditions. Addition of 1 mM Pi did not affect the oxidative stress response. Values indicate average zone of inhibition diameter ± standard deviation from three replicate experiments. Only representative images are show. (b–c) wild type, ΔphoX, and phoXc strains were grown to mid-log phase, osmotic stress tolerance was determined either on solid media (MH agar) containing 0.17 M NaCl (b) or in liquid media (MH broth) containing 0.25 M NaCl (c). These experiments were performed three times. (TIF) [file pone.0026336.s003.tif]
