# Supplementary material for: Contribution of TAT System Translocated PhoX to Campylobacter jejuni Phosphate Metabolism and Resilience to Environmental Stresses
Source: PLoS One. 2011 Oct 20;6(10):e26336. doi: 10.1371/journal.pone.0026336 (PMC3197622; doi:10.1371/journal.pone.0026336)
Supplement: Table S1 — Bacterial strains and plasmids used in this study. (DOC) [file pone.0026336.s004.doc]

**Table S1. Bacterial strains and plasmids used in this study.**

| **Strain/Plasmid** | **Relevant description** | **Source/Reference** |
| --- | --- | --- |
| *C. jejuni* 81-176 WT | Wild type strain of *C. jejuni* | Dr. Qijing Zhang |
| *∆phoX* | *C. jejuni* 81-176 derivative with deletion in *phoX* gene; *phoX*::Kan | This study |
| *∆ppk1* | *C. jejuni* 81-176 derivative with deletion in *ppk1* gene; *ppk1*::Kan | [40] |
| *∆tatC* | *C. jejuni* 81-176 derivative with deletion in *tatC* gene; *tatC*::Kan | [16] |
| *phoX^c^* | *C. jejuni* 81-176 *phoX* mutant complemented with wild type copy of *phoX* with its RBS | This study |
| *E. coli* DH5α | *E. coli* strain used for cloning | Invitrogen |
| pZero-1 | Cloning vector for making suicide vector; Zeo | Invitrogen |
| pZero1-*phoX* | pZero-1 containing the upstream and downstream sequences of *phoX*. | This study |
| pZero1-*∆phoX*-kan | pZero1-*phoX* with *phoX* gene replaced by the pUC4K kanamycin gene through inverse PCR | This study |
| pUC4K | Source plasmid for kanamycin resistance gene | Amersham |
| pRRC | Homologous recombination vector used for *phoX* complementation | [37] |
| pRRC- *phoX* | Suicide plasmid used for insertion of wild type copy of *phoX* back into *phoX* mutant through homologous recombination | This study |
