# Supplementary material for: Contribution of TAT System Translocated PhoX to Campylobacter jejuni Phosphate Metabolism and Resilience to Environmental Stresses
Source: PLoS One. 2011 Oct 20;6(10):e26336. doi: 10.1371/journal.pone.0026336 (PMC3197622; doi:10.1371/journal.pone.0026336)
Supplement: Table S2 — Oligonucleotide primers used in this study. (DOCX) [file pone.0026336.s005.docx]

Table S2. Oligonucleotide primers used in this study.

| **Name** | **Sequence (5’-3’)** |
| --- | --- |
| **Primers used for construction of *∆phoX* mutant and *phoX^c^* complemented strains** | |
| PhoX F | AACCTTGGTACCGATGTTTTACAAGCT AGAGTAGG |
| PhoX R | AAAGACTCGAGGG ACAAATTACCTCAAGTTCTG |
| PhoX INV F | CCTTAGAATTCACCTTTGCTTCATAACCTTGTG |
| PhoX INV R | GGTTGAATTCGGGATTGCTTTTAGTGAGGATT |
| PhoX COMP F | ATTGGATCCGTCATTTATACCTAGTGAAAAC |
| PhoX COMP R | ATTGGATCCTTAGCTTCCTATCACTCCAC |
| **Primers used for quantitative RT-PCR** | |
| *csrA* F | TTATCGGAGAAGGTATAG |
| *csrA* R | TTTCTAAGTATCATAAGGG |
| *spoT* F | GTAACCACTCGCACAATATC |
| *spoT* R | GATGTCGCAGTTTATTCTCC |
| *pstS* F | CCTTATACAAACTGGAATCAAATC |
| *pstS* R | GACACATCACTCATTACAAGC |
| *pstC* F | CGCTTATGCTTTAGGTATGAC |
| *pstC* R | GCTGCCATCACCACTATC |
| CJJ81176_0750 F | GGTCTTGTTGCCTTATTG |
| CJJ81176_0750 R | GTATCGCTATGTTCTATGC |
| *ppk-2*- F | ATCTAATACTCCAACTTGTC |
| *ppk-2*- R | TTCTTCTTCTCCACTACG |
| *ppk-1*- F | TGAAGCAAGTATGGAAGGAG |
| *ppk-1*- R | ATATAGGAGTCATAAGTTCTAAGC |
| *rpoA* F | ATTACAACATCTGCTTATACG |
| rpoA R | TCTACTATTTCTTTATTTGATTCG |
| *aph*C0298 F | GATTATTGGTATTAGTCCTGATAG |
| *aphC*0298 R | AAGTAGAACGAATGATGCC |
| *aphC*0356 F | AGTAATTGGAATTTCAGG |
| *aphC*0356 R | TAAATCATTAACCACAGC |
| *sodB F* | TTATCAAAGGTGCTACAGGAG |
| *sodB* R | CAAACATCTACAACAAGTAAAGG |
| *proP F* | TTACTAATGGTTCTTCCTAC |
| *proP* R | CTTGACAATGTTCTCTTAC |
| *katA F* | CAGTAGCAGGTGAAGCAGGTG |
| *katA* R | GCGGATGAAGAATGTCGGAGTG |
| *16sRNA* F | GTCTCTTGTGAAATCTAATG |
| *16sRNA* R | GTATTCTTGGTGATATCTAC |
| CJJ_0379 F | TAACGCACTTAGCAAGACATTC |
| CJJ_0379 R | GGTATCCTCTACGACGAACTG |
| *CJJ_1374 F* | ACCGCCAATACCATTATG |
| *CJJ_1374 R* | ACTAAGTTCATTACCAAATCC |
| *CmeC F* | GCTGCTGCTCAATTAGGTATAG |
| *CmeC R* | GCTTCATAATCATACTCACTTGC |
